# Dimpled elastic sheets: a new class of non-porous negative Poisson’s ratio materials

**DOI:** 10.1038/srep18373

**Published:** 2015-12-16

**Authors:** Farhad Javid, Evelyne Smith-Roberge, Matthew C. Innes, Ali Shanian, James C. Weaver, Katia Bertoldi

**Affiliations:** 1 Harvard John A. Paulson School of Engineering and Applied Sciences, Harvard University, Cambridge, Massachusetts, 02138, USA; 2 Siemens ADGT, 9545 Cote de Liesse, Dorval, Québec, H9P 1A5, Canada; 3 Wyss Institute for Biologically Inspired Engineering, Harvard University, Cambridge, Massachusetts, 02138, USA; 4 Kavli Institute, Harvard University, Cambridge, Massachusetts, 02138, USA

## Abstract

In this study, we report a novel periodic material with negative Poisson’s ratio (also called auxetic materials) fabricated by denting spherical dimples in an elastic flat sheet. While previously reported auxetic materials are either porous or comprise at least two phases, the material proposed here is non-porous and made of a homogeneous elastic sheet. Importantly, the auxetic behavior is induced by a novel mechanism which exploits the out-of-plane deformation of the spherical dimples. Through a combination of experiments and numerical analyses, we demonstrate the robustness of the proposed concept, paving the way for developing a new class of auxetic materials that significantly expand their design space and possible applications.

The Poisson’s ratio, *ν*, defines the ratio between the lateral and axial strains in a material under uniaxial loading. Theoretically, in linear isotropic materials, the Poisson’s ratio can range between −1 and 1/2. A material with *ν* = 1/2 shears easily and resists volumetric deformations due to its vanishing shear (*G* = 0) and infinite bulk (*K* → ∞) modulus. Conversely, a material with *ν* = −1 resists shear (*G* → ∞), but easily undergoes volumetric deformations (*K* = 0). Outside this range, either the shear or bulk modulus of the material is negative, which is impossible due to thermodynamic stability^1^.

Although the traditional belief is that the Poisson’s ratio of elastic materials must be positive (so that they shrink/expand laterally when stretched/compressed axially), since 1980s many 2D and 3D structures and materials with negative Poisson’s ratio have been reported^2^,^3^,^4^,^5^,^6^. Auxetic behavior was first realized in 2D re-entrant honeycomb structures that unfold and expand laterally when uniaxially stretched^7^,^8^. The same concept was later exploited by Lakes to design and fabricate the first 3D polymeric foam with isotropic auxetic behavior^9^. Subsequently, a number of geometries were proposed to achieve negative Poisson’s ratio through rotation of the stiffer components in the microstructure. These include chiral honeycombs^10^,^11^, networks of rigid rotating units^12^,^13^,^14^,^15^, and elastomeric porous structures in which instabilities are exploited to trigger the rotation of stiff domains^16^,^17^,^18^. Finally, negative Poisson’s ratio was realized in non-porous systems either by embedding an auxetic network within a compliant matrix^19^,^20^ or by using angle-ply laminates^21^,^22^,^23^,^24^,^25^,^26^.

Till now, the majority of materials designed to have negative Poisson’s ratio are porous and this significantly limits the potential applications of auxetic materials. Although low porosity auxetic sheets comprising an array of elongated holes have been recently designed^15^, porosity is still crucial for inducing negative Poisson’s ratio in these systems and, hence, their auxetic response disappears if made non-porous. Auxetic composites can overcome this limitation due to their non-porous structure. However, since their response highly depends on the contrast between the material properties of their different phases, a limited set of engineering materials and manufacturing techniques can be used for their fabrication, making them unsuitable for many industrial applications. Here, we introduce a new class of auxetic materials that are non-porous and are easily fabricated out of any elastic sheet using conventional manufacturing techniques.

## Dimpled Elastic Sheets

**Figure 1:**
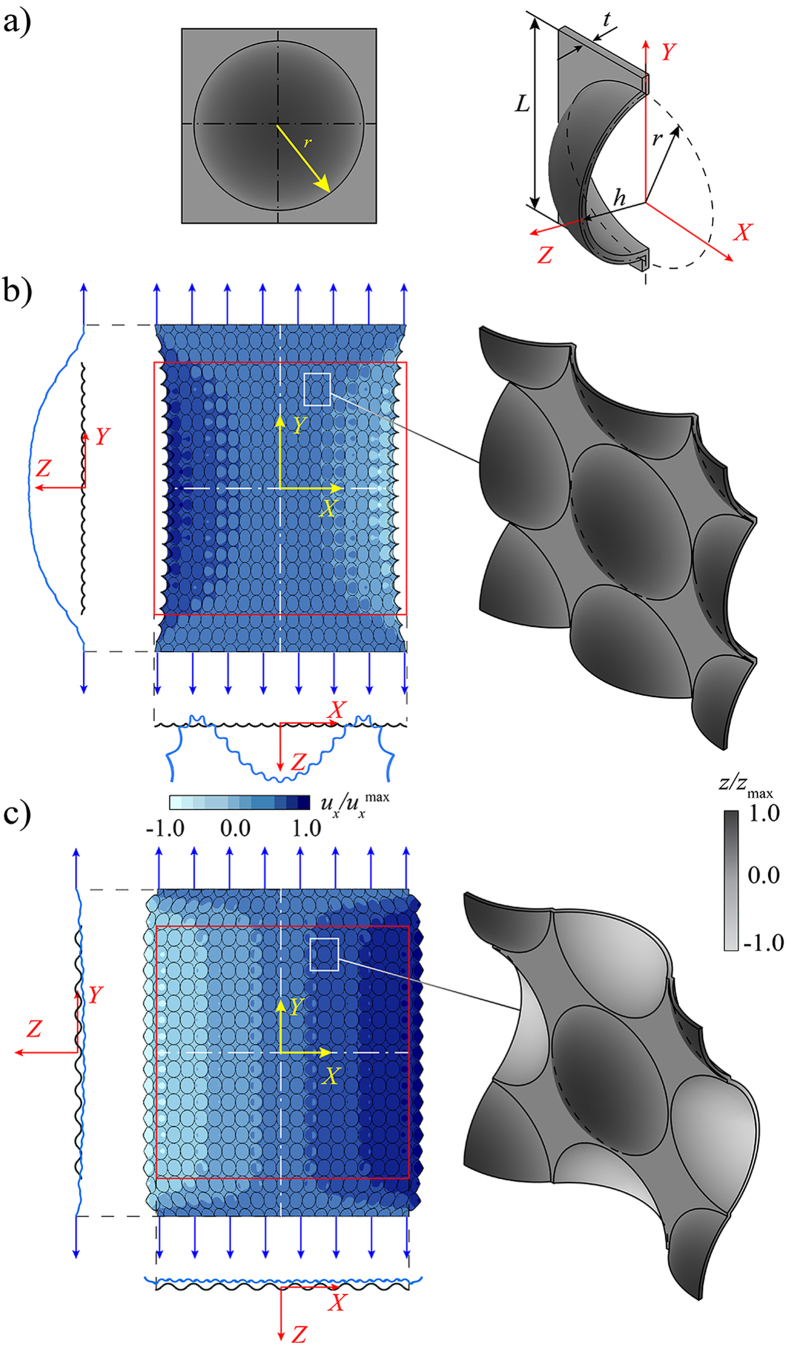
Deformation mechanism of dimpled elastic sheets. (**a**) The building block of the proposed auxetic material comprises a flat sheet dented with a spherical dimple. (**b**) Numerical results showing the deformation under uniaxial tension of an elastic sheet with a square array of 20 × 20 dimples dented on one side of the sheet (we assume *ψ* = 75%, *h*/*r* = 0.5 =  and *t*/*r* = 0.08). The analysis reveals that this structure bends out of plane and contracts laterally. (**c**) The out-of-plane bending of the structure can be prevented by denting the dimples on both side of the flat sheet to form a checkerboard pattern. Such structure expands when stretched and, thus, is characterized by negative Poisson’s ratio. Note that the results shown in (**b**,**c**) have been magnified 600 times to better visualize the deformations.

As shown in Fig. 1a, the building block of the proposed material consists of a square flat sheet with edge *L* and constant thickness *t* dented with a spherical dimple of height *h*. Each dimple is a sector of a thin spherical shell which forms a circle of radius *r* when intersecting the flat sheet. The geometry of the dimpled elastic sheets is then characterized by three dimensionless parameters: the dimple aspect ratio, *h*/*r*, the normalized thickness, *t*/*r*, and the dimple density,


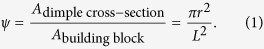


To realize a 2D auxetic material, we arrange the dimples on a square lattice and investigate the response of the system both numerically and experimentally.

We study the behavior of the dimpled structures numerically using the commercial finite element (FE) package ABAQUS/Standard (Simulia, Providence, RI) and investigate the response of the system under uniaxial tension (see **Numerical techniques** in the **Methods** section for details of the FE analysis). We first focus on an elastic sheet with all dimples dented on one side (see Fig. 1b-right). In particular, we choose *h*/*r* = 0.5 and *ψ* = 75% and consider a finite-size domain comprising a square array of 20 × 20 dimples. For such structure, we find that the applied uniaxial stretch causes out-of-plane bending (see side views in Fig. 1b). Moreover, the contour map for the horizontal component of the displacement (*u*_*x*_), shown in Fig. 1b-left, indicates that the system contracts laterally, resulting in a positive value of the in-plane macroscopic Poisson’s ratio (i.e. 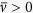). However, it is important to note that the out-of-plane bending of the structure can be suppressed by balancing the system in *z* direction. This is achieved by denting the dimples on both sides of the flat sheet to form a checkerboard pattern (see Fig. 1c-right). Remarkably, in such balanced structure, all dimples flatten toward the structure mid-plane under an applied uniaxial tension, resulting in lateral expansion of the system (see Fig. 1c-left) and, therefore, an auxetic response (i.e. 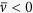). Finally, we note that, although the results reported in Fig. 1 are for a sheet comprising a square array of 20 × 20 dimples, the response of the system is not affected by the finite size of the structure (see Supplementary Information for details).

## Results

### Numerical models

**Figure 2:**
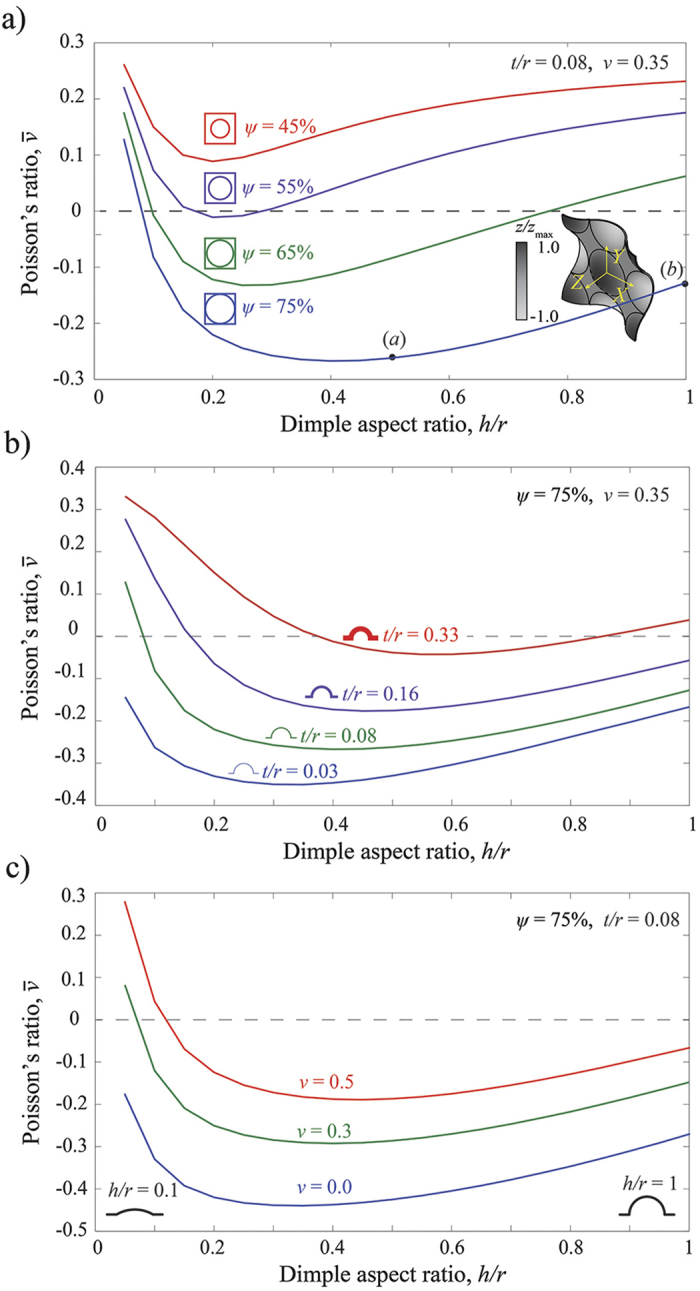
Effect of *h*/*r*, *ψ*, *t* and *ν* on the macroscopic Poisson’s ratio of the dimpled elastic sheet, 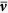. Evolution of 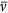 as a function of *h*/*r* is shown in (**a**) for four different values of *ψ* (assuming *t*/*r* = 0.08 and *ν* = 0.35), in (**b**) for four different values of *t*/*r* (assuming *ψ* = 75% and *ν* = 0.35), and in (**c**) for four different values of *ν* (assuming *ψ* = 75% and *t*/*r* = 0.08).

Having demonstrated that an elastic sheet with spherical dimples dented on both sides can exhibit auxetic behavior, we now numerically investigate the effect on the macroscopic Poisson’s ratio of such structure, 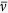, of the dimple aspect ratio, *h*/*r*, the dimple density, *ψ*, the thickness of the elastic sheet, *t*/*r*, and the Poisson’s ratio of the bulk material, *ν*. To ensure the results are not affected by the boundary effects, we focus on an infinite periodic system and study the response of a 2 × 2 unit cell (see inset in Fig. 2a) with periodic boundary conditions^27^,^28^. We stretch the unit cell uniaxially by applying a homogenized strain in vertical direction, 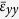, and measure the transverse strain in horizontal direction, 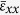. The Poisson’s ratio of the dimpled sheet is then calculated as 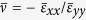. In Fig. 2a, we report the evolution of 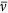 as a function of *h*/*r* for different values of *ψ* (45–75%), while *t*/*r* and *ν* are kept constant. First, the results indicate that, as *h*/*r* increases, 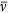 initially drops, reaches a minimum value and then increases. Moreover, we find that the macroscopic Poisson’s ratio monotonically decreases as the dimple density increases and eventually becomes negative. Interestingly, for *t*/*r* = 0.08 and *ν* = 0.35, all structures with *ψ* ≥ 55% exhibit auxetic behavior for a range of dimple aspect ratios, demonstrating that by controlling *ψ* and *h*/*r* dimpled structures with 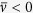 can be designed. Furthermore, we find that the response of the system is moderately affected by *t* and *ν*. In fact, the data reported in Fig. 2b,c show that the auxetic response of the dimpled sheet can be enhanced by decreasing the structure thickness and the Poisson’s ratio of the bulk material. Finally, we should note that, although the results reported in Fig. 2 are for uniaxial tension, we expect the Poisson’s ratio of the structure to remain identical under compressive loading, since we are exploiting a linear elastic effect.

### Experiments

**Figure 3:**
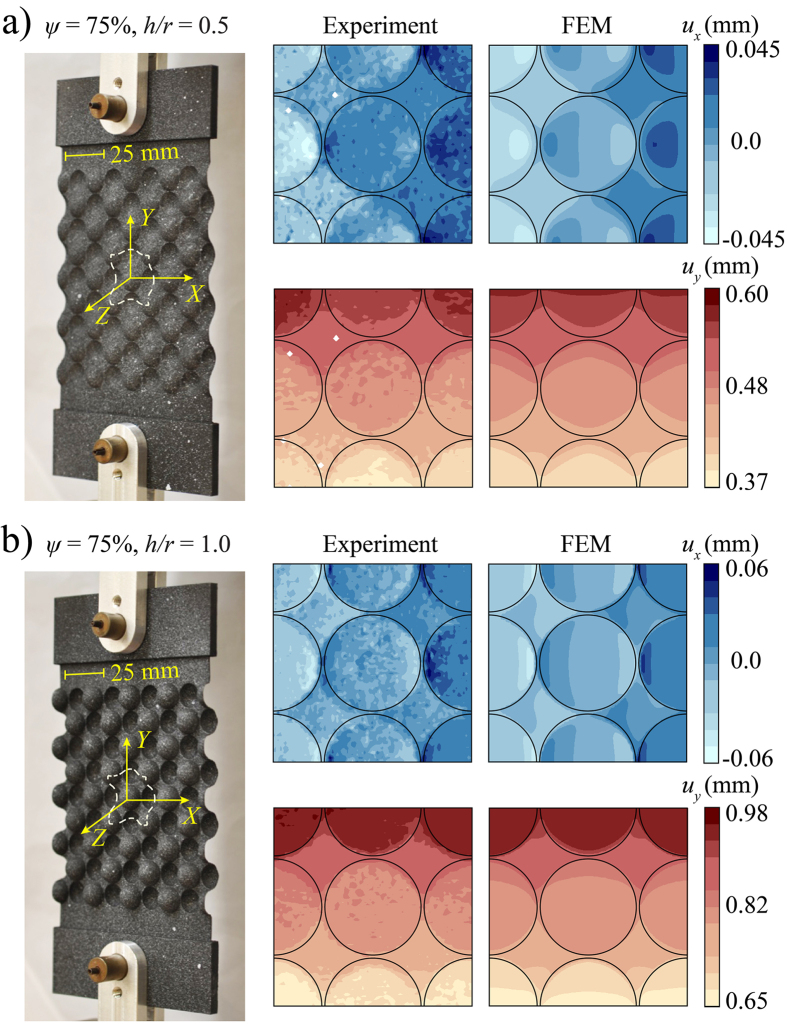
Contour maps for the horizontal (*u*_*x*_) and vertical (*u*_*y*_) components of the displacement fields. Experimental (left) and numerical (right) results are quantitatively compared for two structures characterized by (**a**) *ψ* = 75%, *h*/*r* = 0.5 and (**b**) *ψ* = 75%, *h*/*r* = 1.0. Snapshots of the deformed samples are shown on the left, with the central unit cell highlighted by white dashed lines.

We proceed to verify the numerical predictions experimentally. Two specimens are fabricated using a 3D printer (Connex 500 available from Objet, Ltd.) from VeroClear material (product number: RGD840) with Young’s modulus *E* = 1.5 GPa and Poisson’s ratio *ν* = 0.35 (see Supporting Information for the mechanical properties of VeroClear material). Each specimen comprises an array of 9 × 9 dimples (with *L* = 12.5 mm). In particular, we focus on two structures characterized by (*a*) *h*/*r* = 0.5 and (*b*) *h*/*r* = 1.0 (highlighted by circular markers in Fig. 2a), while we keep *ψ* = 75% and *t*/*r* = 0.08 fixed. The specimens are tested under uniaxial tension in an Instron testing machine and the displacement fields are visualized using a digital image correlation (DIC) technique^29^,^30^. In Fig. 3, we report the experimental contour maps for the horizontal (*u*_*x*_) and vertical (*u*_*y*_) components of the displacement fields obtained for a grip displacement of *u*_grip_ = 1.0 mm (Fig. 3a - resulting in a longitudinal strain of 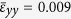 in the central part of the sample) and 1.625 mm (Fig. 3b - resulting in a longitudinal strain of 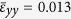 in the central part of the sample). To minimize the boundary effects, we focus on the central unit cell (highlighted with white dashed lines in Fig. 3a,b at left) and compare the results with the numerical predictions for the corresponding infinite periodic structure, showing a very good agreement. Importantly, the contour maps for *u*_*x*_ show that both structures expand laterally when uniaxially stretched, confirming their auxetic behavior.

Next, we use the experimentally measured displacement fields, shown in Fig. 3, to quantify the macroscopic Poisson’s ratio of the dimpled sheets. We first calculate the average displacement components along each of the four boundaries of the central unit cell, 〈*u*_*x*_〉^*R*^, 〈*u*_*x*_〉^*L*^, 〈*u*_*y*_〉^*T*^, and 〈*u*_*y*_〉^*B*^ (*R*, *L*, *T*, and *B* denoting the right, left, top, and bottom edges of the unit cell, respectively), from which the average strain values for the unit cell are obtained as


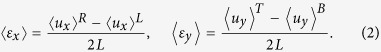


Finally, the macroscopic Poisson’s ratio is calculated as


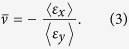


For the two specimens characterized by *h*/*r* = 0.5 (Fig. 3a) and *h*/*r* = 1.0 (Fig. 3b), we find that 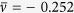 and −0.126, respectively, in excellent agreement with the FE predictions for the corresponding infinite periodic structures (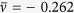 and −0.128). Note that we also simulate the response of finite-size structures (see Supplementary Information for details) and find that the results are very close to those obtained for the infinite domains, indicating that the effect of the boundaries is not pronounced.

## Discussion

**Figure 4:**
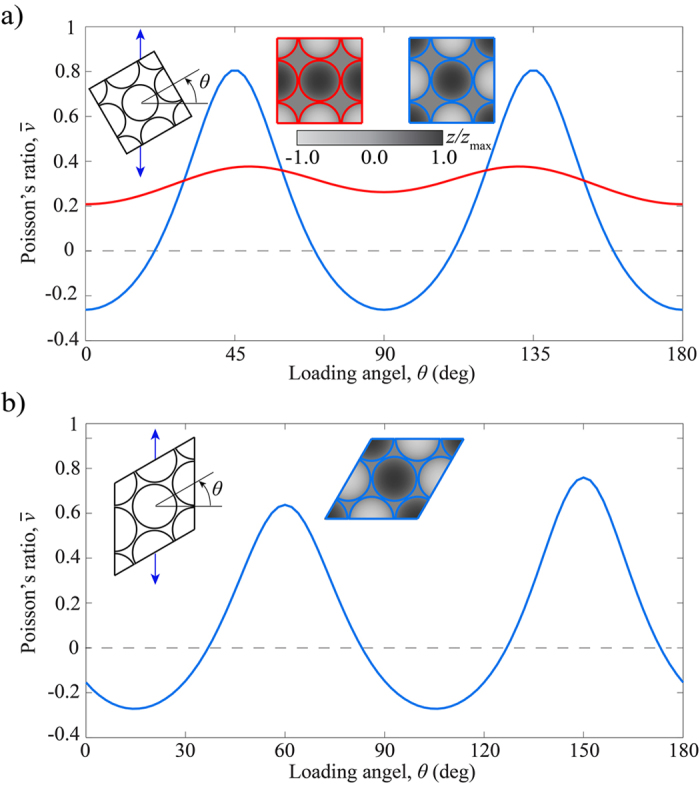
Effect of loading direction, *θ*, and dimples arrangement on 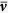. (**a**) Evolution of 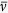 as a function of *θ* for an elastic sheet with a square array of dimples dented to form a checkerboard (blues line) and a stripe (red line) pattern. For this set of simulations, we assume *ψ* = 75%, *h*/*r* = 0.5, *t*/*r* = 0.08 and *ν* = 0.35. (**b**) Evolution of 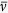 as a function of *θ* for an elastic sheet with a triangular array of dimples dented on both sides. Here, we assume *ψ* = 86%, *h*/*r* = 0.5, *t*/*r* = 0.08 and *ν* = 0.35.

In the previous section, we focused on dimples arranged to form a square array and showed that having a balanced number of them dented on both sides of a flat sheet is a crucial condition to achieve auxetic behavior. However, it is important to realize that both the direction of the applied load, *θ*, and the arrangement of the dimples affect the macroscopic Poisson’s ratio of the sheets. Focusing on the effect of the loading direction, in Fig. 4a we report the evolution of 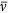 as a function of *θ* for a structure with dimples arranged to form a checkerboard pattern (blue line). These numerical results indicate that the dimpled sheet is characterized by 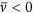 only when loaded approximately in horizontal (i.e. *θ* ~ 0°) and vertical (i.e. *θ* ~ 90°) directions. Large positive values of 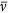 are instead found for *θ* = 45°. In Fig. 4a, we also report results for a structure in which the dimples are still dented on both sides of the flat sheet, but to form a stripe pattern (red line). Although this system is also characterized by an equal number of dimples dented on each side of the sheet, no auxetic behavior is observed, demonstrating the important role played by the arrangement of the dimples. To further confirm this point, we investigate the response of a flat sheet with a triangular array of dimples dented on both sides (inset in Fig. 4b - note that this is the only triangular arrangement characterized by an equal number of dimples dented on both sides of the sheet.). As shown in Fig. 4b, the balanced triangular array of dimples is characterized by auxetic behavior for specific loading directions. In particular, for the considered structure characterized by *ψ* = 86%, *h*/*r* = 0.5 and *t*/*r* = 0.08, 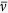 is negative for 174° < *θ* < 36° and 84° < *θ* < 126° and reaches a minimum (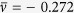) for *θ* ~ 15° and 105°.

**Figure 5:**
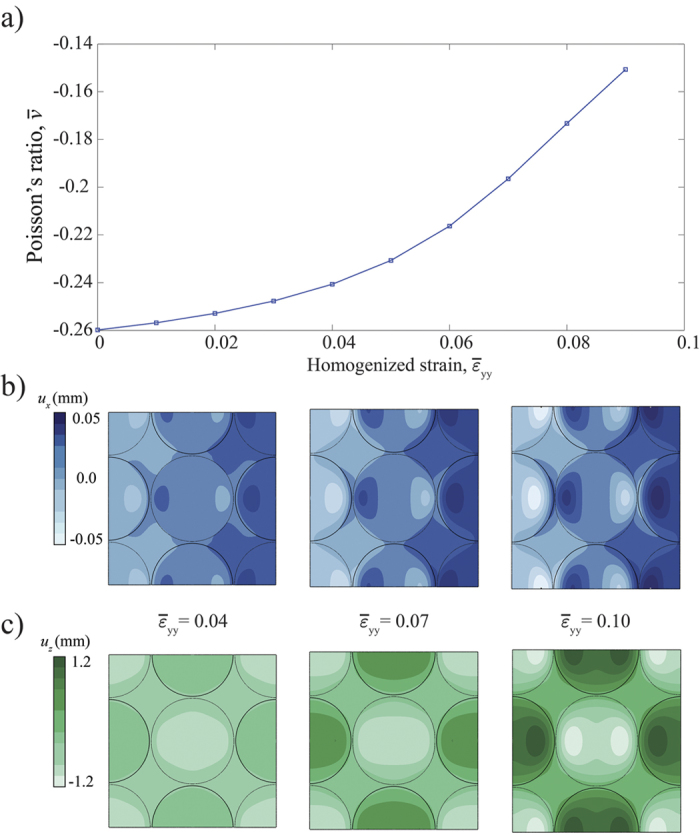
Effect of large applied deformations on the macroscopic Poisson’s ratio of the dimpled elastic sheet. (**a**) Numerical results showing the evolution of 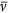 as a function of 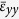. (**b**,**c**) Contour maps for (**b**) the in-plane lateral (*u*_*x*_) and (**c**) the out-of-plane (*u*_*z*_) components of the displacement fields at different levels of strain, 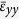.

Finally, it is important to note that 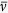 is also affected by the magnitude of the applied deformation. To highlight this point, we numerically evaluate the evolution of the macroscopic Poisson’s ratio, 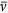, under large deformations (we consider values of 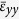 up to 0.1 and conduct a non-linear FE analysis) for an infinite dimpled sheet characterized by *ψ* = 75%, *h*/*r* = 0.5, and *t*/*r* = 0.08. As shown in Fig. 5a, by increasing the applied strain the macroscopic Poisson’s ratio of the structure monotonically increases (i.e. the auxetic behavior weakens). This is because as 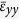 increases, the dimples gradually flatten (see contour maps for *u*_*z*_ in Fig. 5c) and their ability to push the flat sheet in the lateral directions diminishes (see contour maps for *u*_*x*_ in Fig. 5b).

In this study, we demonstrated a fundamentally new approach to generate non-porous periodic materials with negative Poisson’s ratio by denting a homogeneous and uniform elastic sheet with an array of dimples. Through a combination of numerical analysis and experiments, we showed that the Poisson’s ratio of the system can be easily tuned and altered by controlling the arrangement of the dimples. In particular, we found that having an arrangement with a balanced number of dimples dented on the two sides of the flat sheet is crucial to generate auxetic behavior. Importantly, the system we explored can be easily fabricated and has a robust behavior, pointing to a novel and practical method for producing non-porous negative Poisson’s ratio materials.

## Methods

### Numerical techniques

The numerical analysis are performed using the commercial FE package ABAQUS/Standard (Simulia, Providence, RI). The response of both finite-size and unit cell dimpled sheets are investigated under uniaxial tension throughout this work. All models are generated by quadratic tetrahedral elements (ABAQUS element type C3D10M). We should note that we also built FE shell models, but we found that the stress distribution around the dimple edges is not accurate, unless a very refined mesh is used which significantly increases the computational time. In all simulations, we model the response of the bulk material as linear elastic with Young’s modulus *E* = 1.5 GPa and Poisson’s ratio *ν* = 0.35. Moreover, since we focus on the linear response of the structure, we do not account for non-linearities in the simulations.

Finite-size models (see Fig. 1b,c) are initially studied to verify the auxetic behavior in dimpled structures, while the unit cells models (see Figs 2 and 3) are used to ensure the qualitative results are not affected by the boundary effects. In finite-size structures, the uniaxial loading in vertical direction is applied by fixing all nodes on their top and bottom surfaces in *x* and *z* directions and uniformly displacing them in *y* direction while in unit cell models, the loading is applied as a homogenized strain in vertical direction, 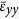. To this end, periodic boundary conditions are applied on all lateral edges of an infinite periodic structure of a 2 × 2 unit cell^27^,^28^.

### Experiments

**Figure 6:**
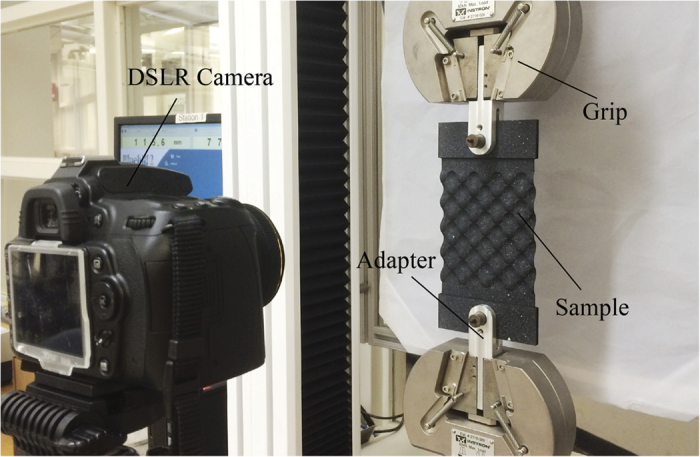
Experimental setup: the camera, the sample, and its connection to the Instron machine are identified on the figure.

Picture of our experimental setup is shown in Fig. 6. An Instron uniaxial testing machine with a 50 kN load cell is used for applying uniaxial tension to the samples. The samples are connected to the machine using two custom-made adapters. To improve the samples alignment, pinned grips are used for connection. A uniaxial vertical displacement (*u*_*y*_) is applied to the upper grip of the Instron machine, while the lower grip is fixed. To quantify 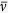, the in-plane deformation of the samples is monitored by taking images at different levels of applied displacement using a high-resolution digital camera (Nikon D90 camera with a 50 mm f/2.8 lens). The images are then analyzed to quantify the in-plane deformation of the specimens using a digital image correlation (DIC) package^29^,^30^. To increase the images contrast, the samples are first coated in black and then speckled with a white spray paint (Krylon Products Group, Cleavland, OH) prior to the test. The speckle pattern generates a density of approximately 2–9 pixels per speckle which, given our experimental setup, leads to a displacement accuracy of 800 nm^31^.

A total displacement of 2 mm is applied to each sample at the low rate of 0.25 mm/min to ensure quasi-static conditions (note that the contour maps shown in Fig. 3a,b are obtained for an applied grip displacement of *u*_grip_ = 1.0 and 1.625 mm, respectively). The sample, the adapters to connect the samples to the grips, the grips, and the camera are all identified on Fig. 6. Two large stand lamps are also used to better light the samples during the tests, which are not shown in Fig. 6.

## Additional Information

**How to cite this article**: Javid, F. *et al.* Dimpled elastic sheets: a new class of non-porous negative Poisson's ratio materials. *Sci. Rep.* **5**, 18373; doi: 10.1038/srep18373 (2015).

## Supplementary Material

Supplementary Information
