# Supplementary material for: Dimpled elastic sheets: a new class of non-porous negative Poisson’s ratio materials
Source: Sci Rep. 2015 Dec 16;5:18373. doi: 10.1038/srep18373 (PMC4680965; doi:10.1038/srep18373)
Supplement: Supplementary Information [file srep18373-s1.pdf]

Supplementary Information for
Dimpled elastic sheets: a new class of non-porous negative Poisson's ratio materials

Farhad Javid,[1] Evelyne Smith-Roberge,[1] Matthew C. Innes,[2] Ali Shanian,[2] James C. Weaver[3] and Katia Bertoldi[1,4*]

[1]*Harvard John A. Paulson School of Engineering and Applied Sciences, Harvard University, Cambridge, Massachusetts, 02138, USA.*

[2]*Siemens ADGT, 9545 Cote de Liesse, Dorval, Québec, H9P 1A5, Canada.*

[3]*Wyss Institute for Biologically Inspired Engineering, Harvard University, Cambridge, Massachusetts, 02138, USA.*

[4]*Kavli Institute, Harvard University, Cambridge, Massachusetts, 02138, USA.*

**Corresponding Author. Tel: +1 617 496 3084; E-mail: bertoldi@seas.harvard.edu*

Additional Experimental Results

Mechanical properties of VeroClear material. All samples tested for this study are fabricated out of VeroClear material using a 3D printer (Connex 500 available from Objet, Ltd.). Since the glass transition temperature of VeroClear is $T_{GT} = 52 - 54$°C, the material is in the glassy phase when tested at room temperature. This is confirmed by a uniaxial tensile test conducted on a flat (non-dimpled) dog-bone shape sample with a testing section of height $\times$ width $\times$ thickness $=$ $87.5 \times 50 \times 0.5$ mm. During the test a maximum tensile displacement of $u_{\text{grip}} = 1.53$ mm (resulting

in a nominal strain of $\varepsilon_{\text{nominal}} = u_{\text{grip}}/\text{height} = 0.017$) is applied to the sample using an Instron uniaxial testing machine and the displacement field is visualized using a digital image correlation (DIC) technique (see the **Methods** section of the main text for details).

In Figs. S1a and b we report the experimental contour maps for the horizontal (u_x) and vertical (u_y) components of the displacement fields, which are used to estimate the Poisson's ratio of the bulk material, ν. In particular, to minimize the boundary effects, we focus on a square region in the center of the sample (highlighted with black dashed lines in Fig. S1a and b) and use the the average displacement components along each of its four boundaries to calculate ν (see **Experiments** in the **Results** section of the main text for more details). Using this procedure we estimate the Poisson's ratio of VeroClear material to be $\nu = 0.33 - 0.38$ (we choose $\nu = 0.35$ for our FE simulations).

Also, in Fig. S1c we show the nominal stress (i.e. the applied load divided by the cross-sectional area at the testing section) versus the nominal strain (i.e. the grip displacement divided by the initial height of the testing section) curve obtained during the uniaxial tensile test. The data clearly show that the material is characterized by a linear behavior at room temperature. By comparing the experimental data to that of finite-size FE simulations we estimate the Young's modulus of the material to be $E = 1.5$ GPa.

Displacement maps. While in the main text we only show the contour maps for the central unit cell (see Fig. 3) and compare the results with the numerical predictions for the corresponding infinite periodic structures, in Figs. S2 and S3 (a-d) we include the contour data generated for

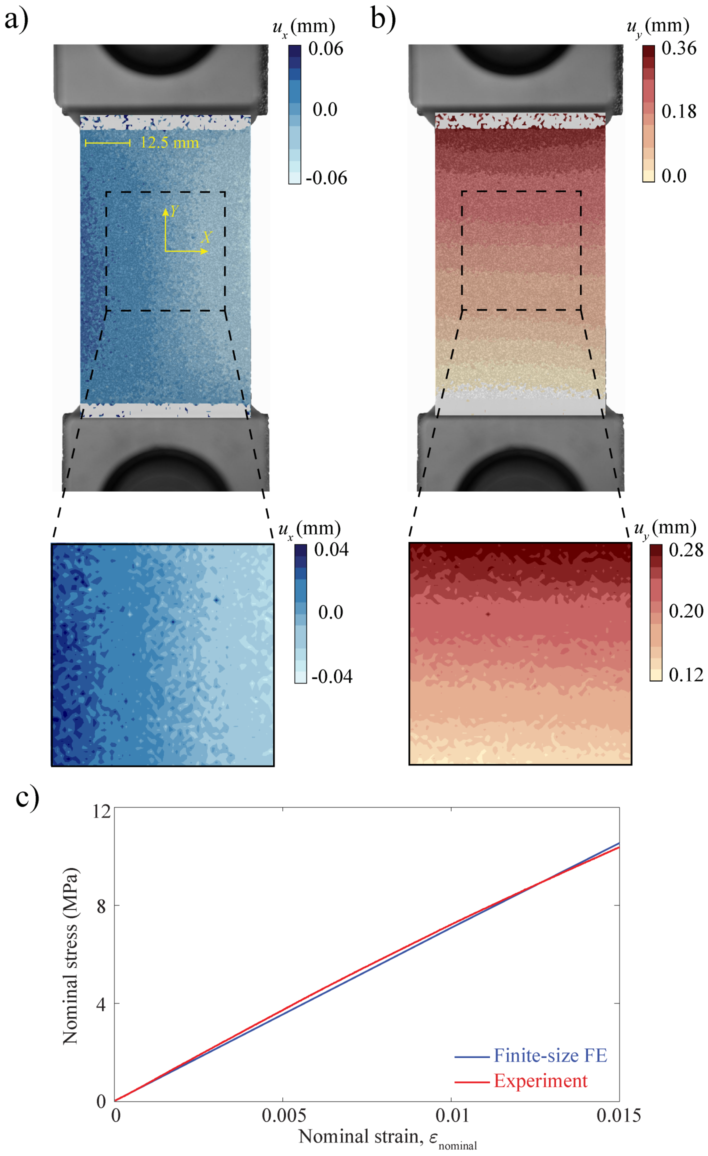

Figure S1: Mechanical behavior of VeroClear material: (a-b) Contour maps for the horizontal (u_x) and vertical (u_y) components of the displacement fields. (c) Comparison of the experimental and numerical stress-strain curves. The data confirm that VeroClear can be modeled as a linear elastic material in the range of small deformations.

the entire samples. In particular, in Fig. S2 we focus on sample (a) characterized by $\psi = 75\%$ and $h/r = 0.5$ at an applied vertical displacement of $u_{\text{grip}} = 1$ mm (resulting in a nominal strain $\varepsilon_{\text{nominal}} = 0.007$), while in Fig. S3 we report results for sample (b) characterized by $\psi = 75\%$ and $h/r = 1.0$ at an applied vertical displacement of $u_{\text{grip}} = 1.62$ mm (resulting in a nominal strain $\varepsilon_{\text{nominal}} = 0.012$). Also, the numerical and experimental stress-strain curves are compared in Figs. S2e and S3e for (a) and (b) structures, respectively. The almost linear stress-stain behavior observed here confirms that no instabilities occur during the tensile tests.

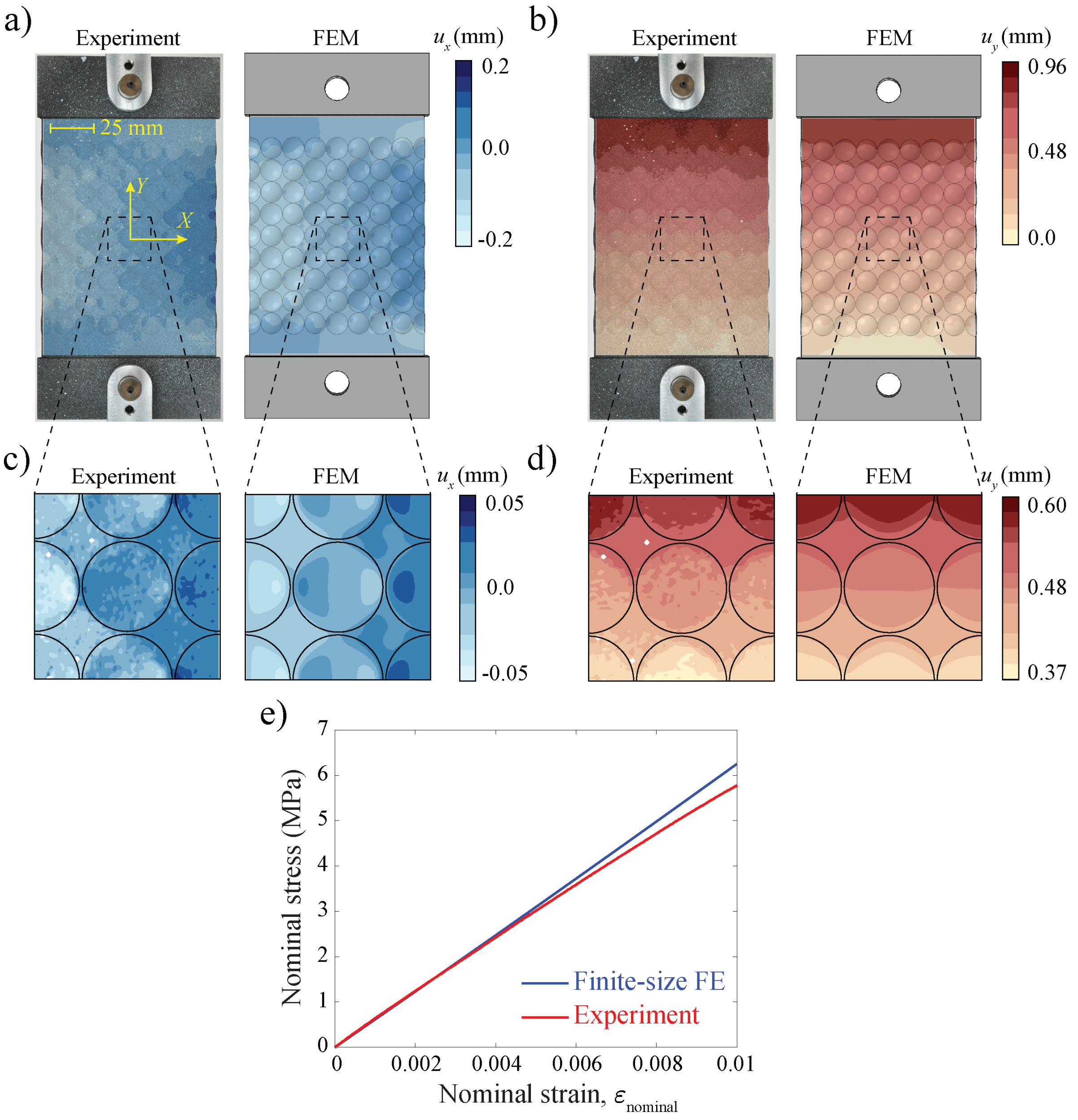

Figure S2: (a-b) Contour maps for the (a) horizontal (u_x) and (b) vertical (u_y) components of the displacement field for the sample characterized by $\psi = 75\%$ and $h/r = 0.5$. Experimental (left) and numerical (right) results are quantitatively compared. (c-d) Zoom-in views of the contour maps for the central unit cells. (e) Experimental and numerical results showing the nominal stress versus the nominal strain up to a tensile strain of 0.01.

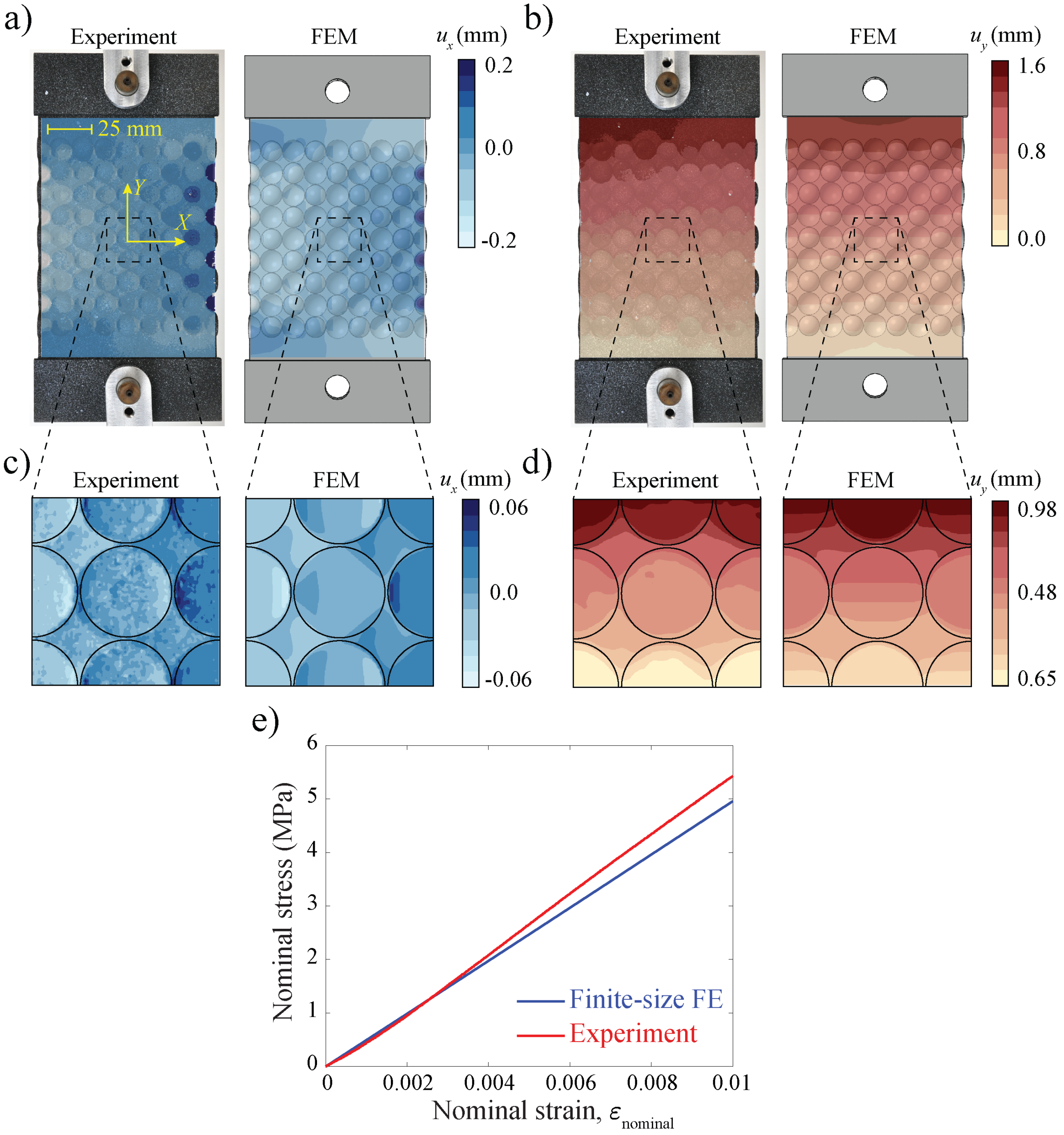

Figure S3: (a-b) Contour maps for the (a) horizontal (u_x) and (b) vertical (u_y) components of the displacement field for the sample characterized by $\psi = 75\%$ and $h/r = 1.0$. Experimental (left) and numerical (right) results are quantitatively compared. (c-d) Zoom-in views of the contour maps for the central unit cells. (e) Experimental and numerical results showing the nominal stress versus the nominal strain up to a tensile strain of 0.01.

Additional Numerical Results

Size effects. In Fig. 1 of the main text we report numerical results for a dimpled elastic sheet comprising a square array of 20×20 dimples with $h/r = 0.5$ and $\psi = 75\%$. We show that, if all dimples dented on one side, the applied uniaxial stretch causes out-of-plane bending and results in a positive value of the macroscopic Poisson's ratio (see Fig. 1b). Differently, if the dimples are dented on both sides of the flat sheet to form a checkerboard pattern (see Fig. 1c), all dimples flatten toward the structure mid-plane under an applied uniaxial tension, resulting in a lateral expansion of the system and, therefore, an auxetic response.

While the results reported in the text are for an array of 20×20 dimples, in Fig. S4 we show numerical results for dimpled sheets comprising arrays of 10×10 (Figs. S4a and b) and 30×30 (Figs. S4c and d) dimples. These structures behave identically to that of the sheet with an array of 20×20 dimples presented in the main text, indicating that their response is not affected by the size of the system.

Furthermore, we investigate the role played by boundary effects on the macroscopic Poisson's ratio, $\bar{\nu}$, when the size of the dimple array in a dog-bone shape sample is decreased. In particular, we use FE simulations to model the response of a sheet characterized by $\psi = 75\%$, $h/r = 0.5$, and $t/r = 0.08$ and comprising an array of $N \times N$ dimples (with $N = 9$, 7, 5, 3). From each simulation, we calculate $\overline{\nu}$ using the same procedure introduced to postprocess the experimental results (see **Experiments** in the **Results** section 2 of the main text for more details). The results reported in Fig. S5 indicate that by decreasing the size of the array the negative Pois-

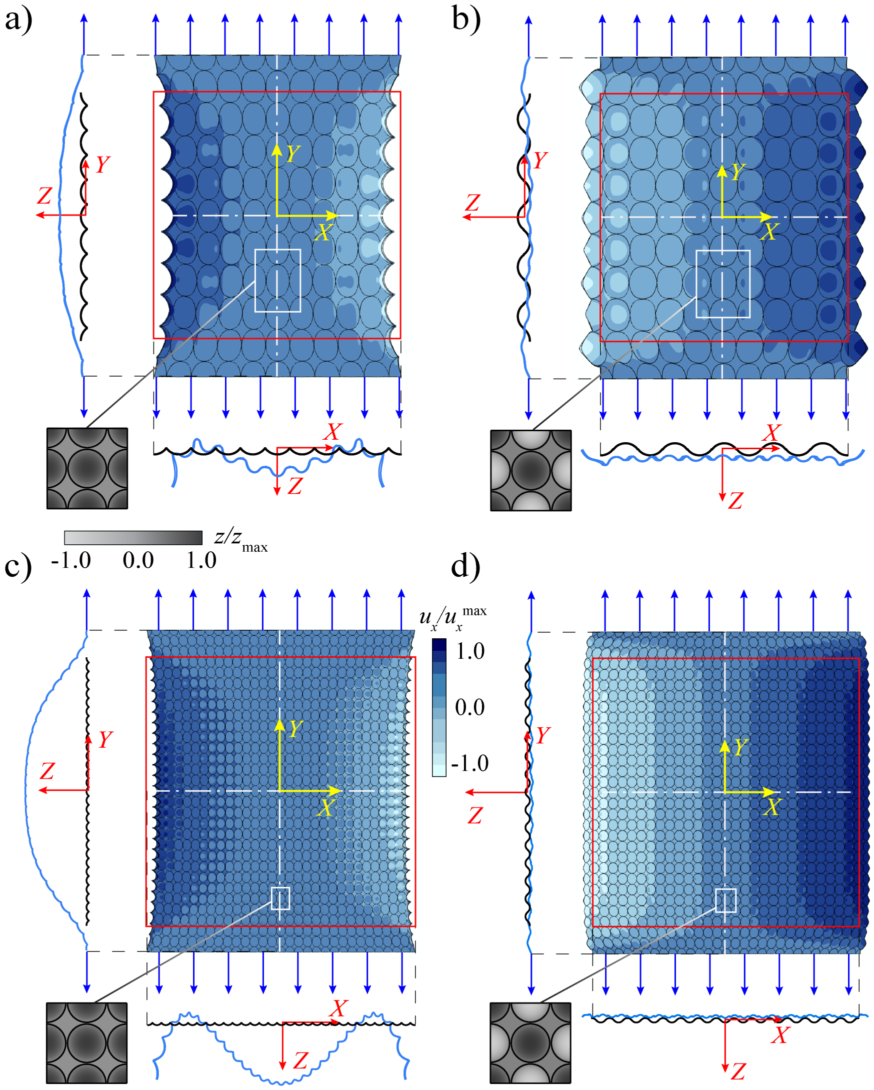

Figure S4: Numerical results showing the deformation under uniaxial tension of elastic sheets with square arrays of (a and b) 10×10 dimples and (c and d) 30×30 dimples. In a and c, all dimples are dented on one side while, in b and d, they are dented on both sides to form a checkerboard pattern.

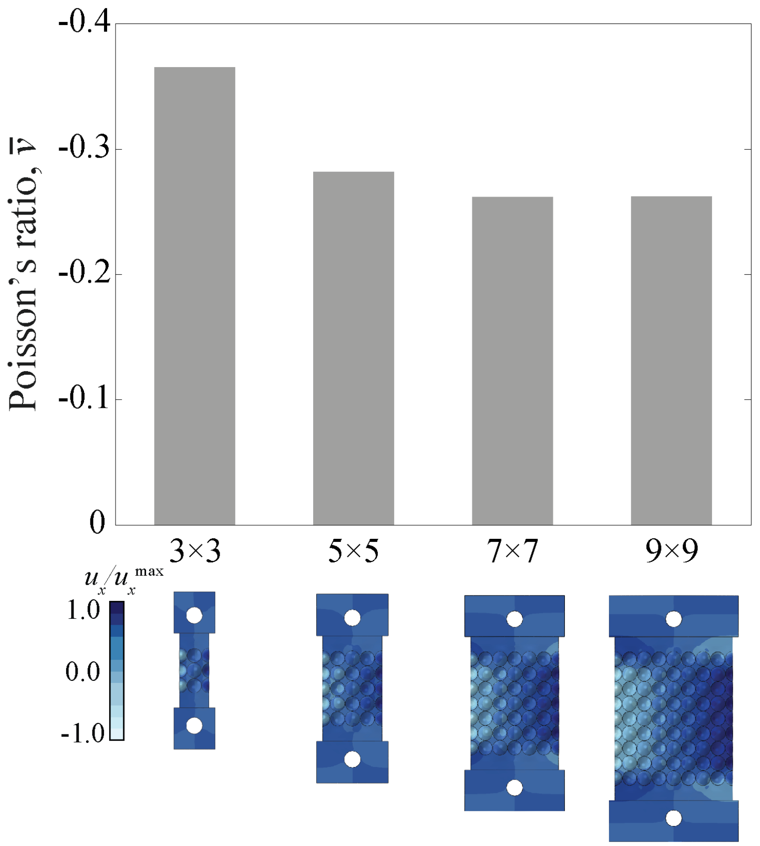

Figure S5: Numerical results showing the Poisson's ratio, $\overline{\nu}$, of elastic sheets with square arrays of $N \times N$ dimples ($N = 9,\ 7,\ 5,\ 3$). The dimpled sheet is characterized by $\psi = 75\%$, $h/r = 0.5$, and $t/r = 0.08$.

son's ratio of the structure becomes more pronounced. This is because the effect of the free lateral boundaries, at which dimples can freely expand, progressively increases.
